# Medical decision-making in hospices from the viewpoint of physicians: results from two qualitative studies

**DOI:** 10.1186/s12904-022-00999-0

**Published:** 2022-09-10

**Authors:** Andreas Walker, Christof Breitsameter

**Affiliations:** 1 ADG Scientific – Center for Research and Cooperation e.V., Albertstraße 3, 56410 Montabaur, Germany; 2 Lehrstuhl Für Moraltheologie, Katholisch-Theologische Fakultät, Ludwig-Maximilians-Universität München, Geschwister-Scholl-Platz 1, 80539 Munich, Germany

**Keywords:** Physician–patient communication, Physician–patient relationship, Palliative medicine, Palliative care, Hospice care, Palliative sedation

## Abstract

**Background:**

Physicians who practice in a hospice are responsible for working with patients and nursing staff to develop a medication plan, monitor symptoms and pain, and adjust medication if necessary. In inpatient hospices in Germany, physicians are part of a multi-professional approach, but not part of the hospice team itself. However, there is no, or hardly any, literature on medical practice in a hospice setting. Therefore, we wanted to know how physicians reflect upon their role in hospice within a multi-professional setting, how they communicate with patients, relatives, nursing staff and other physicians, and what the limitations of these communication processes are.

**Methods:**

By means of two qualitative studies we explored how physicians classify their activities as part of the hospice organization. The study design followed Grounded Theory procedures.

**Results:**

The physicians named an appropriate interpretation of the patient's wishes as the challenge of everyday practice which can lead to differences of perspective with those involved: with nursing staff, who would prefer an alternative form of medication, with relatives, who do not accept that the patient refuses nutrition, with other physicians, who have a different opinion about appropriate treatment. For physicians, this is all the more challenging as communication with the patient becomes increasingly uncertain due to the patient’s illness. Again and again, medical measures have to be negotiated on several levels.

**Conclusion:**

Multi-professional organizations that have to deal with differences in perspective handle them by clearly distinguishing areas of responsibility, an aspect that physicians also claim for themselves. For physicians the question arises repeatedly whether they have correctly interpreted the wishes of the patient. They must continuously reassure themselves of the patient's wishes and this presents them with communication challenges not only with the patient, but also with the nursing staff and relatives and, more recently, with their colleagues.

**Supplementary Information:**

The online version contains supplementary material available at 10.1186/s12904-022-00999-0.

## Background

Hospice and palliative care in Europe are based on a number of common principles that include "the value of patient autonomy and dignity, the need for individual planning and decision-making and the holistic approach" ([1], p. 283). Ideally, the patient should retain his autonomy, among other things, regarding treatment options. "Patients should be empowered to make decisions if they wish.” [1, ibid] In accordance with a holistic approach, inpatient hospices in Germany offer care for the seriously ill that considers physical and emotional well-being, as well as social-psychological health and the existential-spiritual situation.

The respective individual design of the treatment depends on the needs and requirements of the patients^1^ and is organized in a multi-professional team, with the responsibilities divided among the members. The daily administration of medication and the associated symptom and pain control are the responsibilities of nursing staff. Physicians are responsible for working with patients and nursing staff to develop a medication plan, monitor symptoms and pain, and adjust medication if necessary. In addition, both professional groups can also respond to the emotional, psychological, and spiritual well-being of the patients through conversation, presence and listening—whereby these dimensions are also taken on with varying degrees of intensity by volunteers, pastoral workers, social workers or special therapists. In addition, the social-psychological aspect is also covered in treatment by the inclusion of friends and relatives.

In inpatient hospices^2^ in Germany, physicians are part of a multi-professional approach, but not part of the hospice team itself. The hospice team consists in general of the hospice directorship, the directorship of nursing services, nurses and sometimes of a social worker. In addition, there are volunteers, for example chaplains, psychiatrists, art or music therapists and physiotherapists. When looking at existing literature, it is first of all notable that there is no, or hardly any, literature on medical practice in a hospice setting. Although "conflicts at the end of life" have been researched from the viewpoint of the seriously ill and dying in palliative care in various institutional settings, the focus has only been on interactions with nursing staff and not on medical decisions [2]. Employee and family satisfaction with the palliative care of the terminally ill has also been examined, without however taking into account the perspectives of physicians [3, 4]. When it comes to concrete experiences physicians have had with the terminally ill, these were surveyed in hospitals [5, 6], for example, or the focus was more generally on decision-making at the end of life, as in one study that asked general practitioners and medical students about EOL-care [7]. An exception is a study that explicitly asked about the experiences of hospice staff and volunteers and also includes physicians [8]. Further exceptions are recent studies that explicitly examined the competencies required from physicians working within palliative care [9–11]. This noteworthy gap in research, however, is a somewhat problematic oversight considering physicians are the responsible party for the patient’s medical treatment. The situation in Germany has changed in recent years especially in regard to the professionalization of the medical profession in terms of palliative medicine and pain therapy. Additionally, the medical perspective has increasingly come into focus. Palliative sedation and the discontinuation of nutrition and fluid intake in patients have been communicated and discussed more and more openly, as has the level of dosage of painkillers. Therefore, in this article we have considered two studies from different periods. We wanted to know how physicians speak about (1) the medical decision-making processes in hospices, how they speak about (2) the communication processes that lead to these decisions and (3) what limitations these processes are subject to.

## Methods

The research questions required a qualitative design. Data collection was predominantly based on interviews. Data collection and evaluation of our studies followed the methods of Grounded Theory which was developed by Barney G. Glaser and Anselm L. Strauss [12] and modified by Juliet Corbin and Strauss [13], because it abstains from presupposed theoretical concepts, and therefore does not merely verify hypotheses, but generates them. After the responsible ethics committees had declared the study to be ethically acceptable upon submission of the study protocol,^3^ we conducted a study on perspectives of physicians in hospices from 2013 to 2015 in which we interviewed physicians about their role in decision-making processes, and from 2017 to 2020 we conducted a further study in which we again interviewed physicians about their role in decision-making processes in hospices and – this time also – in palliative wards. For the purpose of this article, however, we only evaluated the statements of the physicians who work in hospices. We evaluated the two studies from different time periods because we wanted to know whether the results of the first study would be validated by the second study and whether there were any noticeable changes.

In the first study the interviews were conducted by the authors and one social scientist (Prof, male, 5 years of experience; PhD, senior researcher, male, 3 years of experience, 1 female, researcher) and in the second study by the authors and two social scientists (Prof, male, 10 years of experience; PhD, senior researcher, male, 8 years of experience; PhD, senior researcher, male, 3 years of experience, female, senior researcher, 2 years of experience). We conducted 15 face-to-face interviews from 2013–2015 with physicians working in hospices, palliative wards or in a palliative network and 16 interviews (15 face-to-face, one over the phone) with physicians from 2018–2019. The selection of the study participants took place from 2013–2015 via the hospices to which we already had contact due to our previous research in North Rhine-Westphalia [14–16], and via the palliative networks located there as well as via palliative networks in Northern Germany, with whom we established contact. From 2017–2018 for reasons of comparison we established contact to further hospices, palliative networks and palliative wards of hospitals in the south, north, east and west of Germany (Baden Wurttemberg, Bavaria, Saxony-Anhalt and Hamburg) within the scope of our subsequent study. The sampling was carried out by the hospices or palliative stations we had researched. Among the study participants were a total of 13 physicians (general practitioners, internists, oncologists) with their own practice, 11 clinic physicians working on palliative wards and 7 physicians (pain outpatient clinic, palliative care team) working in a palliative network. Of the 11 clinic physicians, two were also affiliated with a palliative network. All physicians with their own practice regularly visited hospices to look after patients. 15 of the interviewees were female, 16 male (see supplementary file 1).

For the aspect of research dealt with here (medical decision-making processes in hospices from the viewpoint of the physician) we can only refer to those interviews of physicians who are regularly professionally active in a hospice. In the first study this concerns 12 of 15 physicians (P1 to P13, with the exception of P9) and in the second study 10 of 16 (EPH1 to EPH10)^4^ (See supplementary file 2 for an overview of the sampling). The studies are in accordance with the Consolidated criteria for reporting qualitative research (COREQ) checklist [17].

The research interviews took place in the practices, in the clinics or in the hospices and lasted from 15 min to an hour. They were based on a thematic guideline and took place in a semi-standardized form. We asked the physicians in both studies how they classified their activities as part of the overall hospice structure, how they decided to work in hospice and why they opted for palliative medicine, how their contact with patients, relatives and nursing staff was structured and what they regarded as problematic from a medical point of view. We then discussed the role of the patient's wishes as well as conflicts that occur in practice (see supplementary file 3 and 4). If the physicians did not mention "palliative sedation" on their own, we specifically asked about the topic of "sedation." Member checks were carried out to the extent that the interviewers occasionally spoke to the interviewees to clarify and ensure the validity of their core statements.

The interviews were recorded with a digital recording device after clarification and written consent of the participating persons, and then transcribed verbatim, with anonymization of the data. During the clarification we told about the goals and reasons of the study. For the transcription for the first study, we used the semi-interpretive working transcription (HIAT) method; the transcription was done using the EXMARaLDA computer program. In the second study, we used the computer program f4transcript and the analysis tool MAXQDA.

The categories gathered in the research process (in particular “autonomy/self-determination of the patient/resident/guest,” “decision-making,” “palliative sedation”) were worked out with regard to their theoretical properties and again tested in the field. For example, in the course of the studies it was observed that physicians with different specializations (general practitioners, palliative care physicians) do not always have the same ideas about the "autonomy of the patient," which was then particularly focused on in the subsequent interviews with other participants. In line with *theoretical sampling,* physicians from different fields of activity (pain outpatient clinic, palliative network) were intentionally included in the study in order to ensure a data-guided adjustment of the sample selection. This resulted in nuanced differentiations within the conceptual fields to be investigated. We went through several such data collection cycles with simultaneous evaluation. The coding process (open coding, axial coding, and selective coding) was carried out independently by the authors and the results were characterized by a high level of accordance and interrater reliability. Constant comparison was applied to our study, in which we compared the individual perspectives of the interviewees, which we were able to assign to the categories, with the perspectives of the physicians already recorded. In addition, we were able to compare these perspectives with the perspectives of nurses, so that the theoretical properties of the categories were consolidated. The results were compared and discussed in frequent meetings. The data collection process was ended after no additional categories could be gathered from the interviews and it could be said, in relation to the underlying research questions, that a state of theoretical saturation had been reached.

## Results

### Main medical tasks and communication with patients

In the sense of functional differentiation, physicians are responsible for the medical care of their patients—even though they may also attend to the patients´ social concerns or spiritual needs. The main task for a physician to characterize his actions was “drug therapy” (EPH7, 52).

*“It usually goes like this: I come in, I take a look at the files, if there are requests from the nurses that have been recorded […], but that’s also usually done directly, meaning, I look which nurse cares for my patients, then we briefly discuss what’s new, is there something special, has anything come up, then I go to the patients, look at the patients, talk to the patients and then we mostly decide whether something has to be changed with the medication, or not.” (EPH7, 52–60).*

This scheme: physician acquires information from the nursing staff, looks at files, visits the patient, possibly adjusts medication, was usually confirmed by all physicians. Some pain therapists deviate from this scheme and also take part in patient handover in order to gain a general overview of all patients. In addition, the physicians emphasized that it was not only a question of medical care: “It is also often about life, it is also […] about ideas, it is often less about the medical contexts.” (EPH6, 108–109).

Due to his profession, for the physician, the main focus is on the type and method of (pain) medication for the patient. Here, the correct dosage, the discontinuation or increase of the medication play an important role. “So, I am someone who perhaps thinks at a relatively early stage that medication could be discontinued because in our everyday lives we repeatedly experience that patients receive far too much medication.” (P1, 72–75).

Even though medication pain management is the main axis of action for physicians, it is done in a context-sensitive manner. They seem to be guided not only by medical indications, but also by respect for the mental condition of patients. Therefore, a radical discontinuation of medication is not generally to be recommended:

*“[…] so when they arrive, I don't always discontinue everything, because that indicates such a loss of perspective and hopelessness, but in the next few days you usually notice, yes, that they don't want the medication at all and they realize that now we’re dealing with completely different things than just swallowing medication. And then, of course, we often reduce it.” (P6, 74–78).*

It can also happen that the medication is even increased, “because frequently there hasn’t been any adequate pain treatment or because the shortness of breath or fears have not been taken into account.” (P10, 50–52) Curative medication for prophylaxis, the success of which the patient will probably no longer experience due to his limited life expectancy (lipid-lowering agents, antidiabetics), is rather omitted. Other medications used in intensive care or anaesthesia, opioids and sedatives, are increasingly used in hospice care.

### The patient´s wishes and their limitations

Fundamentally, the objective of the physician is to find out what the patient wants. "Does he want to die painlessly? Does he want to die consciously? Is the fear of dying perhaps the problem that must be solved? … and to try to respect the patient's wish, if possible." (P1, 87–89 and 106–115.) The wishes of the patient, according to the predominant opinion of the physicians, are therefore decisive for the medical action. Thus, medical dosage follows a negotiation practice with the patient. The area of tension depends on how much the patient wants to have his pain relieved and/or how consciously he wants to experience the last days of his life. Consequently, physician expertise does not always guide medical decisions. But the tension can lead to disagreements with patients when they refuse to take the medicine indicated by the physician.

*“It sometimes happens that patients are stingy with their medication and no longer want to take it, for example, even if it leads to an increase in pain. Sometimes you have to explain a little about what it is for, or you have to find out why they don’t want to take the medicine anymore, if it’s about side effects for example.” (P8, 97–103).*

Generally, the wish of the patients is the orientation point in these cases as well. But the patient’s wishes can be limited by the physician. If a patient “has more pain than he absolutely has to bear because of his attitude in dealing with medication, then under certain circumstances I would simply order him to take this and that,” a physician said. (P11, 38–40) Interventions also occur if the patient is suffering so much from the pain due to refusal of medication that he disturbs other patients. We were told about a case in which a patient stopped taking morphine because fatigue was a side effect.

*“She now has cancer for the third time, and she is going to die of it at 36. […] She now wants to reduce the morphine. […] Because it makes her tired. You have to have a conversation and say: ‘What is worse for you now: fatigue or pain? Can you bear the pain when you’re awake? Then you don’t need the morphine. But when she screams and the whole hospice collapses in pain, she has to accept the fatigue of the therapy.” (P4, 240–247).*

So, the tension here is between internal factors in the patient (pain, fatigue) and external factors (disruption of other patients by a patient's behavior) which the physician must weigh against one another. Although the physicians could not “force” anyone (P4, 263) to take anything, in these cases the physicians’ actions show paternalistic traits which cannot be reconciled with the patient's wishes. But this communication process is marked by further limitations.

The opportunity for conversation depends on the mental and physical condition and individuality of the patients: “[…] it depends on how accessible the patients are or how much they want to talk about anything.” Some patients focused on their physical condition, others talked about how they would like to spend the last days of their lives or about their realized or unrealized dreams. The topics ranged “from the purely medical to the very personal” and “[…] it varies from day to day and from patient to patient.” (EPH7, 67–78).

If a physician has found personal access to the patient, the relationship can nevertheless fluctuate. “It is quite possible […] that I believe that I have had quite a good conversation with the patient. The day after, I come back and it’s like nothing ever happened.” (P1, 233–235) The physicians reported these uncertainties to us frequently in their communication with patients. With one patient, for example,

*“It was the case that on the first evening I discussed changes that I thought made sense and that I still think made sense. I didn’t understand that she apparently didn’t really understand me. She said ‘Yes’ to everything and ‘Of course’ and ‘That’s how we do it’, we always decide that together, and the next day she asked for the medication we had discussed in detail and wanted it back.” (P10, 57–62).*

### Palliative sedation

A communicational challenge may be the patient’s request for palliative sedation. Apart from the fact that sedation can mean a temporary attenuation of consciousness up to permanent anesthesia, physicians often refer to indication criteria that serve as a guide for consensus among the patient, relatives, nursing staff and the physician. According to one physician the criteria for a palliative sedation include vomiting or fecal vomiting due to intestinal obstruction or shortness of breath, if these symptoms are not treatable with a specific therapy and the patient is in danger of suffocating. And he added: “There are also psycho-social ones, of course, where there is depression or a desire to die. That’s where we hold back.” (P4, 319–320) On the one hand, the patient's wish for sedation is complied with if physical symptoms justify sedation for the physician, on the other hand, the wish is not necessarily complied with if primarily psychological symptoms are decisive for the patient's statement.

*“Well, that is certainly a decision for palliative sedation. That if someone simply can’t breathe and is in danger of suffocation, then I have no problem at all with that. But if someone says that his concept of autonomy is so violated that he doesn’t want to endure it and therefore wants to be sedated, then I have a big problem.” (P11, 71–75).*

Consequently, there are different points of view among physicians as to how the patient’s desire for palliative sedation should be met.

The patient’s consent to, or a wish for a medical measure therefore does not constitute a one-off decision-making process. The physician may have to ensure herself several times that the patient has understood the medical treatment measures. However, this also means that communicative processes in the hospice setting can be more complex, routines need to be reviewed, differences in perspective must be negotiated and communication processes critically questioned.

Another physician observed a difference with regard to the justification of palliative sedation with other physicians: These would not allow palliative sedation in the hospice as long as the patient's physical symptoms were controlled. Psychological symptoms such as “depression” or experiencing a “dark world” did not indicate palliative sedation. For her, this raised fundamental questions about the definition of the situation and the authority to make decisions. The question arose as to whether a physician can at all assess or evaluate whether a symptom is “manageable” or not. The physician tried to answer this question via joint reflection with the patient. In contrast, the palliative physicians were “very rigorous” and would only allow a “medical reason” to apply, which, however, was not necessarily decisive for this physician. (P8, 126–155).

### Communication and differences in perspectives with other agents

A. *Communication with nursing staff*

Together with the nursing team, the physician draws up a medication plan, which takes into account both the long-term medication and the medication on demand, “[…] and actually always in consensus with the patient and the nursing staff.” (EPH7, 60–61) Another physician explicitly distanced herself from the hierarchical expectation of role of the physician:

*“Basically, of course, one could say that the physician has to say what is to be done, yes, so the nursing staff must act accordingly. But I find that nonsense, because the nursing staff has much greater experience, especially in this area, sees the patient 24 h and then, of course, can make suggestions and say, should we do it this way or that way, and, yes, I think so far that we have always agreed quite well.” (EPH7, 228–233).*

Even though the physicians generally refer to the wealth of experience of the nursing staff, whose advice on medication they would be happy to accept, a palliative physician argued that “there is no team decision in the indication. In the indication everyone stands alone before knowledge and his conscience. And it's a medical decision. That's sometimes difficult for the team to understand.” (P12, 74–77) A horizontal, team-oriented communication level is welcomed by many physicians on the one hand, but on the other hand, despite team orientation, the physicians also refer to indication criteria or to professional medical explanations and thus to vertical hierarchy levels.

A form of dissention with nursing staff can arise, for example, when the dying process leads to symptoms that the nursing team considers worthy of treatment and that are emotionally challenging for them, but that are, from the physician’s point of view, part of the dying process and require no further intervention. There is sometimes “a discrepancy in perception: that the patient does not feel his symptoms to be as severe as the nursing staff […] but often the patients don’t suffer the way the nursing staff does. […] You have to then start the conversation and see who’s actually suffering […]” (P13, 55–56 and 74–75 and 63–64.)

Ultimately, the physician must take responsibility for the medical decisions. She can be advised and guided by the team in order to reach a consensus with them, but she can also act in a team-oriented manner and nevertheless adopt a contrary position, just as he could also ignore their views altogether.

B. *Communication with relatives*

In general, relatives are also involved in the treatment of patients. The physicians repeatedly described dealing with relatives as challenging because relatives, for example, have the impression that the hospice is the wrong place for their residents “if they still have or want to see a therapy option.” (P13, 158) When patients’ signs of life decrease—and this is for example indicated by the discontinuation of nutrition and the reduction of fluid intake—relatives often find it difficult to accept the changed symptom situation. “There are always fears that they are starving to death, and fears that they are dying of thirst.” (P5, 122–123) In particular, dealing with relatives presents physicians with challenges when the patient can no longer be addressed and the patient-related (treatment) wish “was actually the relative’s wish.” (P6, II, 143–144) Physicians are therefore critical of and reserved about the relatives’ wishes when they concern treatment options not indicated by the physicians.

C. *Communication with physicians*

In recent times we have often encountered a particular difficulty in coordinating the different medical views and practices in hospice care. When physicians with varying focus and professional specializations are working simultaneously in the hospice (general practitioners and pain therapists, for example) a resulting overlap in areas of activity occurs. An internist described it this way: “I am responsible for everything except pain. […] At the moment it's a bit like the pain therapists are somehow involved in everything. But that’s not really the idea. Because then I don't have anything to do.” (EPH3, 44–49) The pain therapist working in the same hospice presented her point of view to us.

*“So, the classic conflict is: My colleague […] is an internist. And we've got a patient here who has a lot of edema, thick ascites, and she has called for the use of a few diuretics that we don't usually do. That’s what the general practitioners do. […] That sometimes causes friction, above all because I now I have to say: Hm, the patient is getting too many diuretics, now we actually have to monitor potassium levels. How exactly should I tell the general practitioner that she should do a blood test?” (EPH4, 329–338).*

Problems between physicians arise, on the one hand, from overlapping specialist areas and, on the other hand, from communication deficits between the professional groups involved. These differences could be resolved by increased effort in communication, by clarifying—as one physician stated—among the physicians involved, who is responsible for which area—particularly with regard to psychological “stress disorders” (EPH9, 57).

## Discussion

In hospice care, medical indications often lead to the discontinuation or reduction of medication. Physicians take into account cases in which such a measure seems too drastic because it would deprive the patient of all hope. In addition to the medical indication, physicians also pay attention to the context in which they make their decisions. This is true not only with regard to the patient's value system, which affects the administration of medication, at least as long as the patient is capable of autonomy, but also with regard to the emotional state (hope or fear), which, like the value system, can be regarded as a specific form of a patient's cognitive abilities. Thus, in addition to the instrumental considerations, which relate to the medical indication, the physician's decision could also be said to have a prudential dimension as well, which concerns extra-medical factors. Reasons of prudence suggest that these factors should be included in the physician's judgment.

Even where, or especially where, the physician respects the patient's wishes, a tension often emerges between what is medically indicated and what corresponds to the patient's values or the emotional state already mentioned (for example, when the pain relief recommended by the physician leads to a decrease in consciousness, which the patient does not want, so he rejects the proposed medication). According to our observations, this tension is expressed in two ways: (1) first, it is articulated in the fact that the right strategy is negotiated between the physician and the patient. It may be expressed first, in the physician providing a medication that is not (no longer) indicated because the patient wants it, or second, in the physician not (no longer) providing a medication even though it is indicated because the patient does not want it; (2) second, it is articulated in the physician herself making the trade-off. In case (1) we would speak of "shared decision-making", in case (2) of a paternalistic practice. However, not only internal factors, i.e., the patient's motives, play a role, but also external factors, such as the extent to which the care of other patients is impaired if a patient refuses to take pain medication. Here, the tendency is to proceed paternalistically, i.e., to overrule the patient's autonomy.

Focusing on the patient's wishes has several limitations. (1) We observe paternalistic influences, as just mentioned. (2) The patient's mental state, i.e., his ability to understand and articulate his wishes, and his willingness to communicate also have an influence on the ability to exercise autonomy. Complicating this is the fact that this state can change from day to day, that the patient forgets what he has been told or that he changes his mind. These limitations create uncertainty.

The special case of palliative sedation shows the range among the interviewed physicians between a "broad indication" responding to the patient's wishes or autonomy [18] and a rather paternalistic attitude that only allows a "narrow indication," i.e., solely accepts physical suffering as a reason for permanent and deep sedation.

It is striking and yet unsurprising that physicians take into account not only the patient's wishes but also the opinion of the nursing staff, who, after all, spend considerably more time with the patients. In doing so, they make a medical decision, i.e., combine the horizontal information structure with a vertical decision structure. This also causes discrepancies which can arise between physicians on the one hand and the nursing staff and relatives on the other with regard to medication. We encountered discrepancies among physicians only in the second study, not in the first, which may have to do with the further development of pain therapies.

We observe a high need to justify medical decisions which in turn triggers a need for communication with the parties involved. Everything revolves around the patient’s wishes, not only when the physician communicates directly with the *patient*, but also in dealing with nursing staff, relatives and other physicians*.* Limitations of responding to the patient's wishes result on one hand from uncertainty regarding the mental state of the patient, and on the other from the discrepancies in the assessments of parties involved. But the opinions and attitudes of other actors (nursing staff, relatives and physicians) nevertheless play an important role in the decision-making process. Despite these influences (not least the nonmedical factors), the physician's decision is designated as and justified as a medical decision.

These observations may seem trivial, since the wishes of the patient are already at the center of hospice treatment [19]. Less trivial, however, is the observation of how much the appropriate interpretation of these wishes and their enforcement must be fought for repeatedly [20]. This is done at different levels of interpretation. (1) First, the physician must determine to what extent patients are aware of their condition, which can lead to different assessments of the medical prognosis, for example if patients still expect an improvement in their condition in the hospice. (2) Also, the alignment on the appropriate pain medication from a medical point of view can lead to dissent if patients reject the medication and subsequently burden other patients, relatives and nursing staff with their suffering. Even if trust between physician and patient is regarded as a cornerstone of understanding within the physician–patient relationship [21, 22], it does not in any way prevent communicative uncertainties like misunderstandings or dissents. (3) Medical decision-making in hospices is made even more complex by the fact that physicians have to coordinate their decisions with nursing staff, relatives and other physicians. This can lead to differences in perspectives, as the nurses can point to long professional experience or the physicians to their expertise, which subsequently may also lead to dissent. But there can also be differences in perspective between physicians who have different qualification backgrounds. A pain therapist may judge a medical measure differently than a general practitioner. In the end, dissent is resolved through decision-making accountability, even if this resolution does not always produce satisfactory solutions for all. The patient may be denied sedation for psychological reasons, relatives may find it difficult to tolerate the physician’s advocacy of stopping a patient's nutritional intake, and caregivers may perceive the patient's suffering differently from the physician’s assessment.

### Limitations

In our studies, as presented here, we only considered physicians who work in hospices. How physicians reflect on their medical care in palliative wards has not been taken into account. In addition, the last few years have seen an increasing professionalization of the medical profession through the establishment of specialized outpatient palliative care (SAPV) teams and pain outpatient clinics. This challenges classic medical role models—especially in hospitals when palliative medicine penetrates other areas such as intensive care. Other studies will have to show to what extent physicians in their offices, but also in hospitals are influenced by newer forms of therapy in their decisions at the end of a patient's life, according to which medical parameters and in which way they make decisions.

## Conclusion

For the physicians who work in a hospice the question arises repeatedly whether they have correctly interpreted the wishes of the patient according to the patient, whether the wishes remain unchanged over a certain period of time and to which extent they should and can exert influence on the patient for his own good, based on their knowledge and experience. They have to reassure themself of the patient's wishes, which presents them with communication challenges not only with the patient, but also with the care team and relatives and, more recently, with their colleagues. This also becomes clear when patients ask for palliative sedation due to psychological symptoms, a wish that makes physicians hesitate when clear physical indication criteria are missing.

## Supplementary Information

**Additional file 1: Table1.** Overview of the course of studies/data sources.

**Additional file 2: Table 2.** Sampling of the studies regarding physicians working in hospices.

**Additional file 3.** Interview guide for physicians: Study "Decisions in Hospices".

**Additional file 4. **Interview guide for physicians: Study "On 'dying well'. Actor constellations, normative patterns, differences in perspective".

**Additional file 5.** Medical decision-making in hospices COREQ Checklist.

## Data Availability

The datasets used and/or analyzed during the current study are available from the corresponding author on reasonable request.

## Footnotes

1 Seriously ill people who move into a hospice are often referred to as guests or residents. Physicians usually continue to refer to the guests as patients. We decided to follow the terminology of the physicians.

2 According to § 2 of the framework agreement of § 39a paragraph 1 sentence 4 SGB V (social code V), the general criterion for a patient's eligibility to admission to a hospice is 1. a disease that is far advanced and progresses increasingly without any hope of cure; in addition, 2. the patient regularly requires hospice medical treatment and care that cannot (no longer) be provided on an outpatient basis. The severity of the disease, which leads to death in a very short time (6 months or less to live), must be confirmed by a physician. Since the Hospice and Palliative Care Act, which came into force in December 2015, 95% (90% before that Act) of the costs of a hospice stay are covered by health insurance companies. The remaining costs are financed by donations.

3 The first research project was approved by the Ethics Committee of the Medical Faculty of the Ruhr-University, Bochum (Registration-No.: 3850-10), the second research project was approved by the Ethics Committee of the Medical Faculty of LMU Munich (Az-558-15).

4 Abbreviations: e: expert, p: physician; h: hospice.
