# Supplementary material for: Medical decision-making in hospices from the viewpoint of physicians: results from two qualitative studies
Source: BMC Palliat Care. 2022 Sep 10;21:158. doi: 10.1186/s12904-022-00999-0 (PMC9464388; doi:10.1186/s12904-022-00999-0)
Supplement: Supplementary file 1 — Additional file 1: Table1. Overview of the course of studies/data sources. [file 12904_2022_999_MOESM1_ESM.docx]

**Supplementary file 1**

Table 1: Overview of the course of studies/data sources

| **Study** | **Period** | **Research field** | **Main focus of field research** |
| --- | --- | --- | --- |
| Decision processes in hospices | 2010-2015 | - Participatory observation of patient handover, team meetings and case discussions in 3 hospices; - 18 interviews with full-time staff (2010-2011) | Ethical decision-making in hospice care;  Conflicts and conflict regulation in hospices |
|  |  | - 22 interviews with full-time and volunteer staff in hospices (2012) | Providing spiritual care in hospices |
|  |  | - 15 interviews with physicians (2013-2015) | Ethical decision-making in hospices, conflicts and conflict regulation, the importance of spiritual care in hospices |
| “On ‘dying well.’ Actor constellations, normative patterns, perspective differences” | 2017-2021 | - Participating observation of patient handover, team meetings and case discussions in 5 hospices and in two palliative wards. - 150 interviews with full-time and volunteer staff, residents and patients, relatives and associates, therapists; of these, 16 with physicians. | Recording of differences in perspectives in accompanying the dying  Explication of normative patterns associated with differences in perspective  Organization and professionalization of processes in terminal care |
