# Supplementary material for: Medical decision-making in hospices from the viewpoint of physicians: results from two qualitative studies
Source: BMC Palliat Care. 2022 Sep 10;21:158. doi: 10.1186/s12904-022-00999-0 (PMC9464388; doi:10.1186/s12904-022-00999-0)
Supplement: Supplementary file 2 — Additional file 2: Table 2. Sampling of the studies regarding physicians working in hospices. [file 12904_2022_999_MOESM2_ESM.docx]

**Supplementary file 2**

Table 2: Sampling of the studies regarding physicians working in hospices

| **Study** | **Number of physicians interviews** | **Fields of activity/**  **Age at time of interview** | **Experience with medical practice in an inpatient hospice** | **Frequency of visits/ duration** |
| --- | --- | --- | --- | --- |
| Decision processes in hospices | 12 total, of which 7 male, 5 female | physicians with their own (general practitioner) practice: 7, 4 male, 3 female; 59 to 62 years old (P1, P2, P3, P5, P6, P10, P11) | 6 to 16 years | 1 to 3 per week/ no response |
|  |  | Clinic physicians, physicians who work on palliative wards: 2, male, both also active in a palliative network; 46 and 59 years old (P7, P8) | Several years, up to 18 years | 3 per month or  once every two months/no response  . |
|  |  | Pain outpatient clinic:  2 female; no response and 54 years old (P12, P13) | 2 to 12 years | Twice a week,  4 to 5 pcs. |
|  |  | 1 retired internist/palliative care physician; 72 years old (P4) | 12 years | No response/no response |
| “On ‘dying well.’ Actor constellations, normative patterns, perspective differences” | 10 total, of which 4 male, 6 female | Physicians with their own (general practitioner) practice: 6, 3 male, 3 female; 33 to 64 years old (EPH1, EPH2, EPH3, EPH6, EPH7, EPH9) | 0.5 to 14 years | 1 to 2 per week/ 15 minutes to 2 hours |
|  |  | Pain outpatient clinic, MVZ (medical service center): 3 female; 57 to 64 years old (EPH4, EPH5, EPH10) | 7 to 20 years | 1 to 2 per week/ no response |
|  |  | Palliative Care Team: 1 male; 37 years old (EPH8) | 2 years | 2 per week/30 to 60 minutes per patient |

Abbreviations: E: expert, P: physician, H: hospice
