# Supplementary material for: Medical decision-making in hospices from the viewpoint of physicians: results from two qualitative studies
Source: BMC Palliat Care. 2022 Sep 10;21:158. doi: 10.1186/s12904-022-00999-0 (PMC9464388; doi:10.1186/s12904-022-00999-0)
Supplement: Supplementary file 3 — Additional file 3. Interview guide for physicians: Study "Decisions in Hospices". [file 12904_2022_999_MOESM3_ESM.docx]

**Supplementary file 3**

**Interview guide for physicians**

*Study "Decisions in Hospices"*

**Introduction: relation to the hospice**

Since when have you been visiting a hospice regularly and how often?

How is your practice in the hospice organized?

**How to deal with conflicts**

Do you have any differences in medication with the patient and/or nursing staff? How do you resolve them?

To what extent do you respond to the guest's wishes regarding medication?

What do you think are "moral" or "ethical" decisions in a hospice? How often do these occur? Do these decisions lead to conflicts?

**How to deal with spirituality**

Do guests express wishes to you regarding religiosity/spirituality? If so, in what way can you fulfill these wishes?

Does this lead to conflicts between the needs of the guests and your medical responsibility? How do you resolve these conflicts?

Is your practice different for guests of the Jewish or Muslim faith than for guests of the Christian faith, or does it not matter?

**Summary**

What changes/improvements do you wish for the future regarding communication in a hospice?
