# Supplementary material for: Medical decision-making in hospices from the viewpoint of physicians: results from two qualitative studies
Source: BMC Palliat Care. 2022 Sep 10;21:158. doi: 10.1186/s12904-022-00999-0 (PMC9464388; doi:10.1186/s12904-022-00999-0)
Supplement: Supplementary file 4 — Additional file 4. Interview guide for physicians: Study "On 'dying well'. Actor constellations, normative patterns, differences in perspective". [file 12904_2022_999_MOESM4_ESM.docx]

**Supplementary file 4**

**Interview guide for physicians**

*Study "On 'dying well'. Actor constellations, normative patterns, differences in perspective"*

**Introduction**

Would you please introduce yourself first?

What is your relationship to the hospice/palliative ward? How did your relationship with this institution develop? How often are you here on site?

**Activity**

What do you usually do when you're here?

How can I imagine your work here? What falls within your area of responsibility?

Do you only talk with patients/guests about medical questions or are you also focused on other topics?

Has your work changed since you've been here? Did you do things differently before?

Can you describe a case here at the facility that you think went very well, where you could say "Man could die well"?

Can you also remember a case that you would say was rather problematic or difficult, where some things did not go well?

What do you think is particularly important for the guests/patients here in the facility? What are their main needs and how are these needs met?

Have you ever encountered limits in your work (moral or legal)?

**Contact with staff/relatives**

How is your contact to the other professional groups here in the institution, to the nursing staff or to pastoral care? What do you talk about? Do you sometimes have different views or disagreements?

How do you see the role of the relatives?

**Summary**

What is the special feature/characteristic of this hospice/palliative ward for you?

What would you change if you could? And in any case, what must remain as it is?
